# Effects of Hypoxia and Chitosan on Equine Umbilical Cord-Derived Mesenchymal Stem Cells

**DOI:** 10.1155/2016/2987140

**Published:** 2016-06-09

**Authors:** D. J. Griffon, J. Cho, J. R. Wagner, C. Charavaryamath, J. Wei, A. Wagoner Johnson

**Affiliations:** ^1^College of Veterinary Medicine, Western University of Health Sciences, Pomona, CA 91766, USA; ^2^Department of Biomedical Sciences, College of Veterinary Medicine, Iowa State University, Ames, IA 50011, USA; ^3^Department of Mechanical Science and Engineering, University of Illinois, Urbana, IL 61801, USA

## Abstract

Chitosan opens new perspectives in regenerative medicine as it enhances the properties of mesenchymal stem cells (MSCs) through formation of spheroids. Hypoxia has also been proposed to enhance stemness and survival of MSCs after* in vivo* implantation. These characteristics are relevant to the development of an off-the-shelf source of allogenic cells for regenerative therapy of tendinopathies. Umbilical cord-derived MSCs (UCM-MSCs) offer an abundant source of immature and immunoprivileged stem cells. In this study, equine UCM-MSCs (eqUCM-MSCs) conditioned for 3 and 7 days on chitosan films at 5% oxygen were compared to eqUCM-MSCs under standard conditions. Equine UCM-MSCs formed spheroids on chitosan but yielded 72% less DNA than standard eqUCM-MSCs. Expression of* Sox2*,* Oct4*, and* Nanog* was 4 to 10 times greater in conditioned cells at day 7. Fluorescence-labeled cells cultured for 7 days under standard conditions or on chitosan films under hypoxia were compared in a bilateral patellar tendon defect model in rats. Fluorescence was present in all treated tendons, but the modulus of elasticity under tension was greater in tendons treated with conditioned cells. Chitosan and hypoxia affected cell yield but improved the stemness of eqUCM-MSCs and their contribution to the healing of tissues. Given the abundance of allogenic cells, these properties are highly relevant to clinical applications and outweigh the negative impact on cell proliferation.

## 1. Introduction

Stem cell therapy offers new strategies to manage musculoskeletal conditions that challenge traditional therapeutic approaches, such as spinal cord injury, tendon diseases, chronic inflammation, bone defects, and cartilage damage [1–4]. Among these, tendinopathies have been reported to account for 30 to 50% of musculoskeletal injuries, affecting approximately 100 million human patients globally each year [5]. The avascular nature and limited regenerative potential of tendons contribute to the morbidity of tendon diseases, including slow and incomplete recovery. Tendon diseases challenge traditional medicine and have therefore prompted interest in new alternatives, such as stem cell therapy [1]. As this approach warrants further scientific evidence, tendon injuries in horses are appealing as natural models of tendinopathy in man because of the biological similarity between the equine superficial digital flexor and Achilles tendon in humans [6–8]. Ligament and tendon injuries are present in up to 77% of performance horses, and lameness is the most common cause of wastage in these animals [9–11].

Autologous stem cell therapy has produced some encouraging results in horses with experimental models [12] and naturally occurring tendon injuries [8, 13]. However, this approach remains limited by the morbidity associated with tissue collection, delayed administration due to processing or reprogramming of cells, and the influence of the patient's health status and age on the properties of stem cells [14, 15]. These limitations provide a rationale for investigating allogeneic stem cells as an off-the-shelf alternative. Fetal adnexa-derived cells are appealing candidates, because they circumvent the ethical concerns and risk of teratoma formation associated with embryonic stem cells. Among fetal adnexa, umbilical cord matrix (UCM, also named Wharton's Jelly) provides an abundant source of mesenchymal stem cells (MSCs). These cells have also been found to proliferate faster and over a greater number of passages than amniotic membrane-derived MSCs in horses [16].

Enhancing the potential for self-renewal and multilineage differentiation of MSCs is relevant to large scale cell-banking and serves as a premise for improved therapeutic effects. Chitosan is an aminopolysaccharide derived from shellfish, which is biocompatible and has been used in FDA approved wound dressings and hemostatic agents [17]. We have previously reported on the superiority of chondrogenesis [18–20] and formation of cellular aggregates (spheroids) in contact with this biomaterial [21–24]. Although the exact mechanism of action remains unclear, the formation of spheroids was found to enhance the stemness of adipose- and placenta-derived stem cells in two independent studies [25, 26].

For cell therapy to be effective, cells must also survive implantation, remain local, and contribute to tissue repair. Hypoxic conditioning of stem cells has been proposed as a strategy to achieve these goals, based on the discrepancy between standard culture techniques (normoxia: 19% O_2_) and the physiological hypoxia of native niches for stem cells [27, 28].* In vitro*, the majority of studies reporting improved multilineage differentiation under hypoxia involved bone marrow-derived cells [27], but a few have confirmed these effects on fetal adnexa-derived MSCs [29–31]. However, the impact of acclimating cells to hypoxic conditions* in vitro* on their* in vivo* contribution to the healing of tissues inherently hypoxic remains largely unexplored. Similarly, the combined effects of hypoxia and chitosan on MSCs have not been explored.

The first objective of this study is to determine the effects of conditioning stem cells with chitosan and hypoxia on their* in vitro* properties. We hypothesize that conditioning stem cells improves their stemness. Our second objective is to determine the influence of this conditioning on the healing of tendon defects treated with these cells. We hypothesize that conditioned stem cells are biocompatible, survive after implantation, and improve the healing of experimentally injured tendons over cells cultured under standard conditions.

## 2. Materials and Methods

### 2.1. *In Vitro* Evaluation

#### 2.1.1. Cell Culture

Equine MSCs were isolated with collagenase after harvesting the umbilical cord matrix (eqUCM-MSCs) of six horses during normal foaling. Isolated MSCs were maintained in DMEM media (#SH30021, Hyclone Laboratories Inc., Logan, UT) containing 10% FBS (SH3008803IH, Hyclone Laboratories Inc., Logan, UT) and 1% antibiotics (penicillin (100 U/mL) and streptomycin (100 *μ*g/mL), Hyclone Laboratories Inc., Logan, UT). MSCs were passaged using standard trypsinization protocols at 80–90% confluence. Cells were harvested at P4 and cells from each horse were divided in two groups: Group S (standard) cells were seeded on the standard polystyrene plate (control group; Olympus Multiwell plates, Genesee Scientific, San Diego, CA) and cultured in 19% O_2_, while Group C (conditioned) cells were seeded on the chitosan-coated plate (experimental group) at a density of 5000 cells/cm^2^ and cultured at 5% O_2_ (Incubator Eppendorf Galaxy® 170) at 37°C. Each group was cultured for 3 and 7 days to evaluate growth kinetics and stemness. Cells were cultured for 7 days prior to differentiation and* in vivo* assays.

#### 2.1.2. Chitosan-Coated Plates

Chitosan (deacetylation ≥ 75%, Sigma C3646, St. Louis, MO) was dissolved in 0.67% acetic acid to form a 1% chitosan solution. Plates were coated as published in Cheng et al. [25]. Briefly, 500 *μ*L of chitosan solution was distributed into each well (of 12-well plate) and dried uncovered overnight under the laminar flow cabinet. Acidity of chitosan films was neutralized by 1 mL of 0.5 N of NaOH solution for 2 hours at room temperature, prior to washing plates three times with distilled water. Plates were sterilized by soaking in 70% ethanol overnight in the laminar flow cabinet, after which they were washed with 1x PBS three times. The lids were removed for UV sterilization under the flow cabinet. Prior to seeding, the chitosan films were rinsed with PBS; then fresh media were added.

#### 2.1.3. Growth Kinetics

The growth kinetics were analyzed via DNA content to palliate the inability to count cells due to the formation of spheroids on chitosan films. 5 × 10^4^ cells of P4 were seeded in each well of a 12-well plate for all conditions. Cells were harvested on day 7, lyophilized (−50°C, 0.014 bar), and suspended in 150 *μ*L of 1 mg/mL Hoechst 33258 (Sigma, St. Louis, MO) solution. 30 *μ*L of cell lysate was mixed with 70 *μ*L of TNE buffer in 96-well plate. Salmon sperm DNA was used to determine the standard curve and DNA content was measured by a microplate reader (350360ex/460em, BioTek, Winooski, VT) at day 3 and day 7.

#### 2.1.4. Differentiation Assays

Cells cultured on standard plates were differentiated in the same plate, while changing the media to the appropriate differentiation media and culturing them in an incubator with standard settings (5% CO_2_, 19% O_2_, 37°C). For cells conditioned on chitosan for 7 days, the spheroids were harvested and transferred on a standard plate in specific differentiation media and cultured in an incubator with standard settings.

*(1) Osteogenic Differentiation*. Cells were rinsed with PBS and chitosan spheroids were transferred onto standard plates. Osteogenic media (DMEM/F-12, 10% FBS, 10 mM *β*-glycerophosphate, 10^−7^ M dexamethasone, 0.05 mM ascorbic acid-2-phosphate, and 1% PEST (100 units/mL penicillin; 100 *μ*g/mL streptomycin)) were added to each well and cultured for two weeks. Differentiation was observed by staining with 2% Alizarin red solution.

*(2) Chondrogenic Differentiation*. Standard cells were trypsinized and washed with PBS; chitosan spheroids were collected with a pipette and washed with PBS. Harvested cells were transferred to a conical shaped 96-well and pelleted cells at 200 ×g for 5 minutes. StemPro® Chondrogenesis Differentiation media (Gibco Life Technologies, Carlsbad, CA) were changed three times a week. After three weeks, each micromass was fixed in formaldehyde and stained in Alcian blue for 40 minutes and washed according to standard protocol. Each micromass was transferred onto a glass slide, mounted in 50% glycerol, and imaged for overall integrity of cell mass.

*(3) Neurogenic Differentiation*. Cells were harvested and cultured in an 8-chamber slide culture system (Nunc*™* Lab-Tek*™* II Chamber Slide*™* System, Thermo Fisher, NY). Chambers were coated with polyornithine and laminin prior to transfer. On the day of transfer, standard cells were trypsinized and washed with PBS, and chitosan spheroids were collected by pipetting and washed with PBS. Cells were transferred into each chamber and 200 *μ*L of neuronal differentiation media (Neurobasal A media (Life Technologies #10888022, Carlsbad, CA), 1x B27 (Life Technologies #17504044, Carlsbad, CA), 1 *μ*m retinoic acid (Sigma R2625, St. Louis, MO), and 1% PEST (100 units/mL penicillin; 100 *μ*g/mL streptomycin)) was added. Media were changed three times a week and cultured for 3-4 weeks. To observe differentiation, cells were fixed with 4% formaldehyde for 15 minutes and blocked for 1 hour with blocking buffer (10% normal goat serum in 1x PBS) and then stained with anti-MAP2 (1 : 100, sc-20172, Santa Cruz Biotechnology, Inc., Dallas, TX). Washed cells were mounted in Vectashield with DAPI (H-1200, Vector Laboratories, UK) and imaged with EVOS® fl Cell Imaging System (Life Technologies, Carlsbad, CA).

#### 2.1.5. Stemness

Total RNA was isolated using Direct-zol*™* RNA MiniPrep (Zymo Research, Irvine, CA) after 3 and 7 days of culture. cDNA was synthesized using Superscript®III first strand synthesis supermix (Life Technologies, Carlsbad, CA). qPCR was performed with SYBR®green PCR master mix (Applied Biosystems, CA) on StepOne real-time PCR system (ABI Science, NY). Equine specific primers were used to determine the expression of pluripotent genes* Oct4*,* Sox2*, and* Nanog* with* GAPDH* as the reference gene. Expression levels of individual genes were analyzed by quantitative RT-PCR and normalized to* GAPDH* and expression levels were compared using the comparative CT (2^−ΔΔCT^) method after 3 and 7 days of culture.

#### 2.1.6. Data Analysis

Results were expressed as mean and standard deviation. A paired *t*-test was used to determine if there was a difference in DNA, expression of* Sox2*,* Oct4*, and* Nanog* genes, and magnitude of change across time between the standard and the conditioned cells, within pairs of cell populations from the same horse.

### 2.2. *In Vivo* Evaluation

#### 2.2.1. Study Design

Based on kinetics and stemness assays, conditioned cells were tested after 7 days of exposure to chitosan and hypoxia. Conditioned and standard cells from each horse were transfected with Green Fluorescent Protein (GFP) and expanded. A defect was created in both patellar tendons of rats. One defect was left empty (control) while the other was injected with 0.5 × 10^6^ of labeled cells from Group S or C. Activated rat conditioned plasma (ACP) was used as a carrier for all stem cells. Tensile properties (*N* = 6 rats/treatment, 28 days) were measured in a cyclic, nondestructive mode and normalized to contralateral, untreated defects. Tendons were examined for fluorescence prior to histological scoring of tissue healing (*N* = 4 at day 7 and *N* = 6 at day 28).

*(1) Animals*. All use of animals (horses and rats) was reviewed and approved by our institutional animal use and care committee. Adult male Sprague-Dawley rats (*N* = 25, 4-5 months old, weight 350–375 g, Charles River) were included in the study. Three rats were used to collect blood and produce conditioned plasma used as a carrier for stem cells. The tendons of these rats were harvested after euthanasia and tested biomechanically. The 22 remaining rats were acclimated for two weeks prior to surgical removal of the central third patellar tendon on each limb. One tendon was randomly assigned to a type of labeled stem cells (S or C) while the contralateral defect was left empty, to serve as internal control. Rats were euthanized at 7 days (*n* = 8 rats) or 28 days (*n* = 12 rats) by intracardiac injection of pentobarbital (100 mg/kg) under anesthesia with sevoflurane.

**Figure 1:**
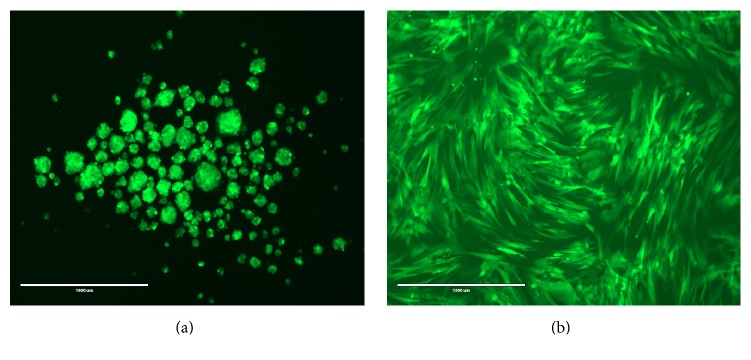
(a) GFP-labeled eqUCM-MSCs cultured on chitosan under hypoxia. Note the formation of spheroids; (b) GFP-labeled eqUCM-MSCs cultured under standard conditions (bar = 1000 *μ*m).

*(2) GFP Labeling of Cells*. Equine UCM-MSCs were transduced with lentivirus containing CMV-GFP expression vector (LP-EGFP-Lv105-0200, GeneCopoeia Inc., Rockville, MD). P2 cells were cultured on 10 cm culture dish in culture media (DMEM + 10% FBS + 1% penicillin and streptomycin) to 70–80% confluence. Media were removed and transduction media (200 *μ*L of LV-CMV-GFP + polybrene (4 *μ*g/mL) + 7 mL of culture media) were added and incubated at 37°C and 5% CO_2_ overnight. Transfection media were removed the next day and replaced with fresh media. After 2-3 days, cells were transferred to T75 plate with culture media containing puromycin (2 *μ*g/mL, high dosage) for stable cell selection up to one week, changing the media every 3-4 days (Figure 1). Transduced GFP expressing MSCs were expanded in culture media and cryopreserved for future purposes.

**Figure 2:**
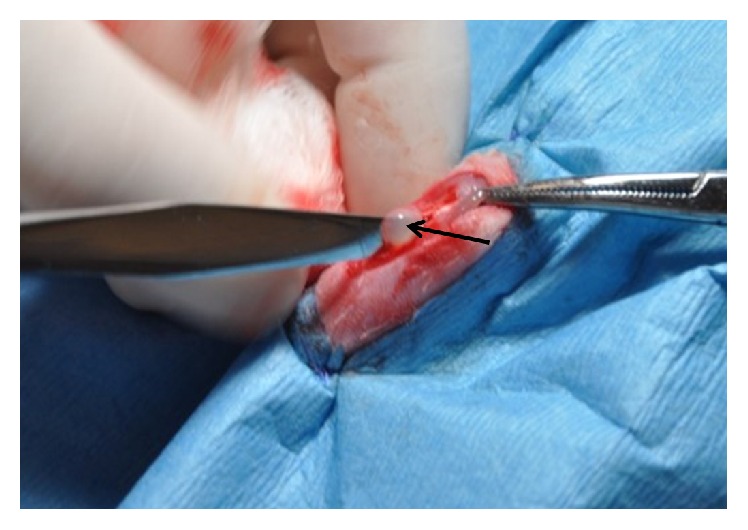
Intraoperative image of a patellar tendon defect treated with stem cells in activated conditioned plasma. The forceps point to the proximal extent of the patellar tendon and the scalpel handle is placed within the defect created in the central portion of the tendon. Note the gelatinous appearance of the ACP (arrow), serving as a carrier for stem cells.

*(3) Activated Conditioned Plasma (ACP)*. 5 mL of blood was collected by cardiac puncture from an anesthetized rat, in a syringe containing 1 mL of acid-citrate-dextrose (5 : 1 v/v; Sigma-Aldrich, St. Louis, MO). The blood was transferred into a vacutainer and centrifuged at 350 ×g for 15 minutes at room temperature. The supernatant portion (yellow) was aliquoted into microtubes and stored at −20°C until use. One hour prior to implantation, 0.5 × 10^6^ cells from Group C or S were added to 100 *μ*L of thawed CP. Activation was achieved by adding 5 *μ*L of 10% calcium chloride (1 : 10 by vol.) resulting in formation of a gel (Figure 2).

*(4) Tendon Injury Model*. Rats were anesthetized with sevoflurane and both stifles were prepared for aseptic surgery. Analgesia was provided with an intramuscular injection of meloxicam (1 mg/kg) after induction. Each patellar ligament was exposed with a craniomedial approach to the stifle. The central 1/3 of each patellar ligament (about 1 mm) was excised from the distal aspect of the patella to the tibial tuberosity [32]. A 0.9 mm diameter Kirschner Wire was used as a template to standardize the size of the defect in each limb. One limb was randomly left untreated (internal control). The contralateral defect was randomly assigned to one of the two treatments: activated CP containing 0.5 × 10^6^ of GFP-labeled eqUCM-MSCs (1) cultured under standard condition or (2) conditioned to hypoxia on chitosan for 7 days (Figure 2). Fascia was closed with a cruciate pattern and skin was closed with an intradermal pattern using 5-0 Polyglactin 910 (VICRYL®, Ethicon, Cincinnati, OH). After surgery, rats received 8 mg of enrofloxacin and 8 mg meloxicam once daily, orally for 7 days.

*(5) Nondestructive Biomechanical Testing*. Patella-tendon-tibia tuberosity units were harvested after euthanasia and observed for gross abnormalities, such as swelling and fibrosis. Soft tissues and ligaments around the stifle were removed except for the patellar tendon. Elastic and viscoelastic properties were measured with a Bose BioDynamic Test system at 37°C equipped with a 10 N load cell. Samples were kept in PBS fluid until just before testing. During the testing, samples were kept moist by spraying PBS fluid on the surface after loading the sample in the clamping fixture. Samples were fixed in position with Bose tension grips, directly clamped on the bone. Tests were conducted in displacement control with a prescribed displacement ramp corresponding to a strain rate of 0.005/s. Samples were unloaded to zero strain at the same rate. Samples were subjected to two load cycles at the prescribed strain rate to 0.5% strain, followed by stress relaxation (ramp to prescribed strain and hold) tests at strains of 0.0025, 0.005, and 0.0075. Only elastic properties were measured because bone-tendon units typically fail at the grips when tested to failure, which invalidates the test. The properties measured included tangent modulus (modulus at a specified strain range, used for nonlinear elastic materials like tendon), hysteresis (energy loss in a cyclic load), and stress relaxation (equilibrium stress under a prescribed strain and corresponding time to reach that stress). The tangent modulus here was calculated at 0.1% strain.

#### 2.2.2. Histopathology

Histopathology was performed on all specimens at 7 days and after biomechanical testing at 28 days. Each tendon was fixed in 10% neutral buffered formalin solution and 9–15 transverse sections (5 *μ*m) from the midportion of each tendon were processed for histology, including H&E and Masson's trichrome staining. Sections were examined to detect the presence of hematoma within the defect as well as pathological changes such as inflammation. An investigator (CC) unaware of the group assignment of each specimen scored each tendon based on collagen grade, cartilage formation, and degree of angiogenesis, using a system previously validated [33]. Scores assigned to each parameter were added to calculate a “histological score.” The histological scores of the three tissue sections were averaged for each specimen.

In addition, 3–5 sections of tissue per tendon were subsequently processed for immunohistochemistry staining. A blocking buffer (10% normal goat serum in PBS) was applied for 1 hour at room temperature prior to overnight incubation at 4°C in 488 fluorophore conjugated GFP antibody (1 : 100, #21311; Life Technologies, Carlsbad, CA). The sections were washed in PBS 4 times for 15 minutes each, mounted in a DAPI (containing mounting reagent (Vectashield, SouthernBiotech, Birmingham, AL)), and examined with EVOS fluorescent imaging system (Life Technologies, Carlsbad, CA). Digital images (10x magnification) of 3 sections per tendon were obtained to determine the average percentage of fluorescent area per field, and the fold changes were compared to those of the contralateral empty defect.

#### 2.2.3. Data Analysis

Results are expressed as means and standard deviations. Significance level was set at 0.05 and *p* values lower than 0.1 were considered as indicating a trend in change throughout the study. Morphometric parameters, biomechanical data, and percentage of fluorescence of each treated tendon (S or C) were compared to those of the corresponding contralateral tendon (including an empty defect) with a paired *t*-test. Histological scores of treated tendons were compared to their untreated contralateral tendon at 7 and 28 days using a linear-mixed effects model with a nested treatment effect. The *t*-statistic was used to test if an effect was statistically significant.

Each tensile property (TP) was normalized to that of the matched contralateral empty defect by calculating the following ratio:

(1) $$\begin{array}{c} \%\text{ of normalized change}=100\times \frac{\left[TP\;\;treated\;\;tendon\left(T1\;\;or\;\;T2\right)/TP\;\;contralateral\;\;tendon\left(E\right)\right]}{\left[TP\;\;contralateral\;\;tendon\left(E\right)\right]}. \end{array}$$

The magnitude of normalized change induced by *T*1 and *T*2 was compared with a paired *t*-test for parametric data and a Wilcoxon signed-rank test for histological scores, pairing samples with similar cell source (same horse). A Wilcoxon signed-rank test was used to determine if there was a statistically significant difference in each histological outcome measure between the 7-day postoperative period and 28-day postoperative period. Fluorescence measured at day 7 versus day 28 was compared with a two-sample *t*-test. Finally, Spearman's correlation coefficients were calculated to determine the correlation between total histological scores, fluorescence, and biomechanical properties of tendons at 28 days.

## 3. Results

### 3.1. *In Vitro* Evaluation

**Figure 3:**
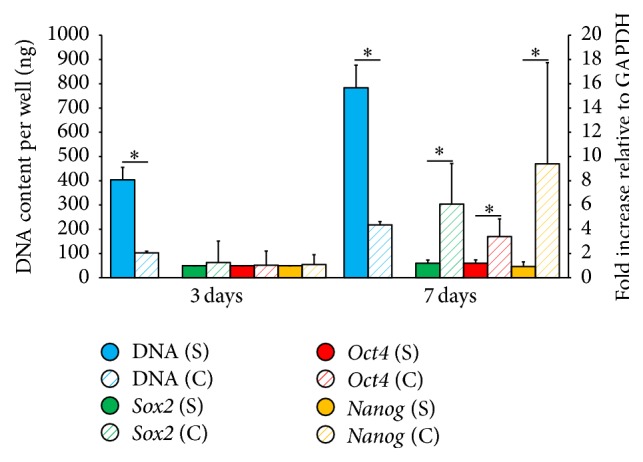
*In vitro* comparison of cells cultured under standard conditions (S) or conditioned on chitosan (C) under hypoxia. Results are expressed as means and SD. *∗* denotes *p* value < 0.05.

**Figure 4:**
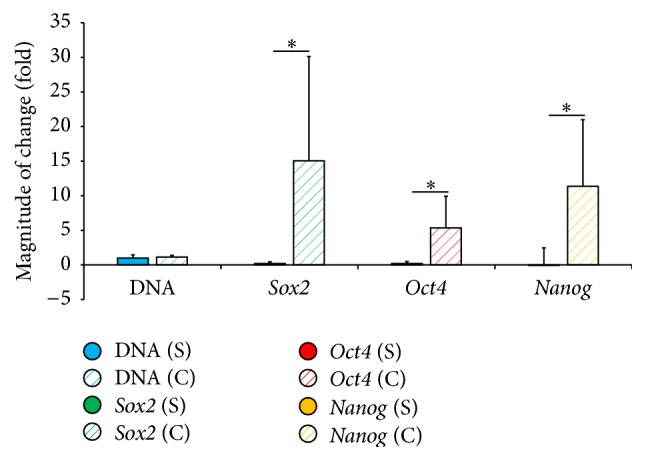
Magnitude of change (mean ± SD) in DNA content and gene expression within each group between day 3 and day 7. Cells were cultured under standard conditions (S) or conditioned on chitosan (C) under hypoxia. *∗* denotes *p* value < 0.05.

**Figure 5:**
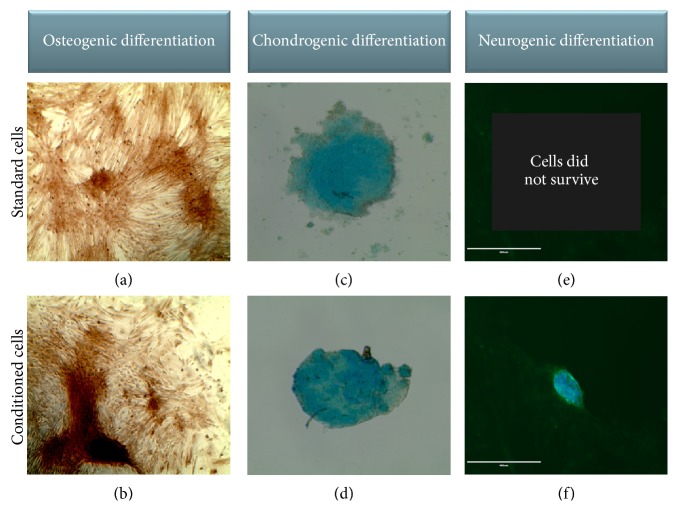
Differentiation of standard (top) or conditioned (bottom) cells (×10): (a, b) osteogenic differentiation (Alizarin red); (c, d) chondrogenic differentiation (Alcian blue); (e, f) only conditioned cells survived and differentiated into neurogenic lineage (*α*-MAP2 and DAPI overlay).

Equine UCM-MSCs formed spheroids on chitosan but yielded 72% less DNA than under standard conditions (Figure 3, Supplemental Table  1 in Supplementary Material available online at http://dx.doi.org/10.1155/2016/2987140). No difference was found in the increase in DNA content detected between days 3 and 7 in both groups (Figure 4, Supplemental Table  2). Expression levels of* Sox2*,* Oct4*, and* Nanog* did not differ at day 3 but were 4 to 10 times greater in conditioned than standard cells at day 7 (Figure 3, Supplemental Table  1). The upregulation of these 3 genes between days 3 and 7 was enhanced when cells were exposed to chitosan and hypoxia (Figure 4, Supplemental Table  2). Equine UCM-MSCs in both groups differentiated into osteogenic and chondrogenic lineages but only conditioned cells underwent neurogenic differentiation (Figure 5). Cells cultured on tissue treated plates did not survive the neurogenic differentiation culture.

### 3.2. *In Vivo* Evaluation

**Figure 6:**
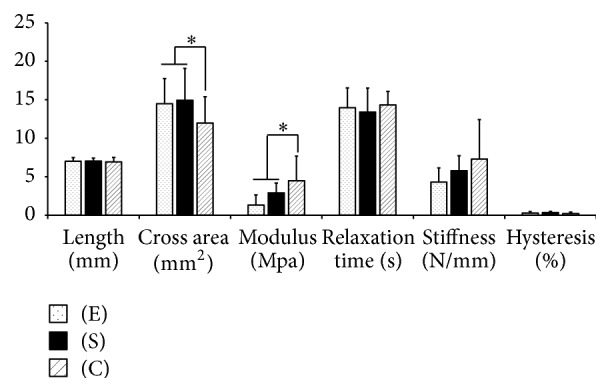
Tensile properties of tendons treated with cells cultured under standard conditions (S) or cells conditioned with chitosan and hypoxia (C) at 28 days. Results are standardized to contralateral tendons with empty defects (E) and expressed as means and SD. *∗* denotes *p* value < 0.05.

The morphometric and tensile properties of tendons are listed in Figure 6 and Supplemental Table  3. No difference was found between the lengths of tendons in all groups but the cross-sectional area of tendons treated with conditioned cells was smaller than that of tendons treated with standard cells (*p* = 0.04). Similarly, the cross-sectional area of tendons treated with conditioned cells was smaller than that of their contralateral untreated tendons (*p* = 0.02). No difference was found between tendons treated with standard cells and their contralateral untreated tendons (*p* = 0.17). Tendons treated with conditioned cells had a greater modulus of elasticity than contralateral untreated defects (*p* = 0.03) and the standard group (176.6% versus 34%, *p* = 0.04). No difference was found between the modulus of tendons treated with standard cells and that of contralateral untreated tendons (*p* = 0.86). A similar trend was identified when stiffness was tested, where tendons treated with C (*p* = 0.06), but not those treated with S (*p* = 0.52), tended to be stiffer than their matched contralateral controls. In comparison, tendons harvested from 3 normal rats had a greater modulus (14.35 ± 1.08 Mpa, *p* < 0.0001) and stiffness (15.34 ± 1.67 N/mm, *p* < 0.005) than any of our 3 study groups (C, S, and E).

**Figure 7:**
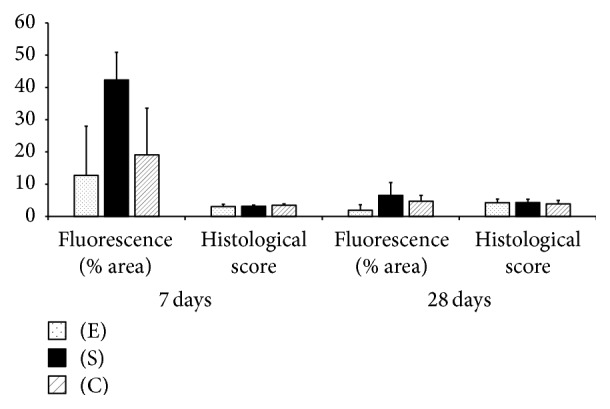
Histological features of tendons treated with cells cultured under standard conditions (S) or cells conditioned with chitosan and hypoxia (C) at 7 and 28 days, standardized to contralateral tendons with empty defects, (E) and expressed as means and SD.

**Figure 8:**
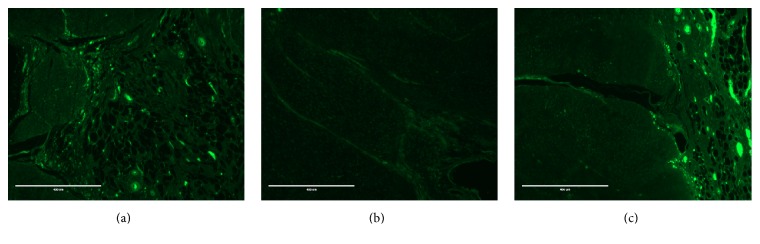
Fluorescence in tendon defects at 28 days: (a) tendon treated with conditioned GFP-stem cells; (b) tendon with an empty defect; (c) tendon treated with standard GFP-stem cells (bar = 400 *μ*m).

**Figure 9:**
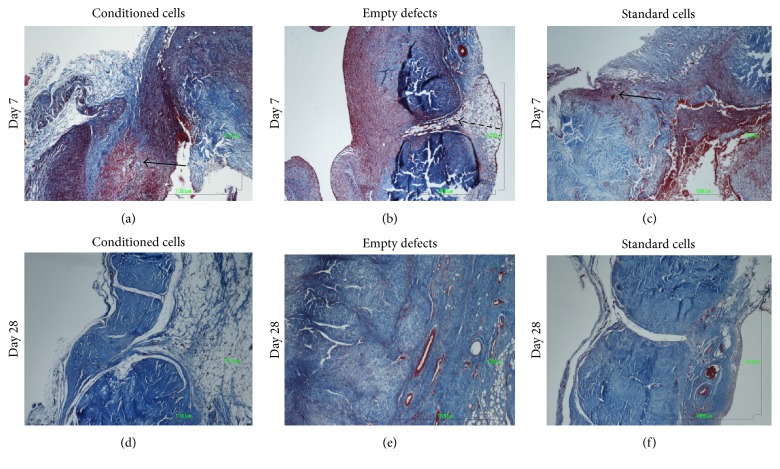
Histology of cross sections at 7 (a–c) and 28 days (d–f). A hematoma is noted in treated defects (a, c, arrows) at 7 days, consistent with the implantation of cells in ACP. The empty defect (dashed arrow) is visible at day 7 (b). Defects are filled with tissue in all groups at 28 days (d, e, f). Masson's trichrome, ×4 (bar = 1000 *μ*m).

Fluorescent cells were identified in all treated defects at 7 and 28 days after surgery and the percentage of fluorescent area was greater in treated defects (C or S) than untreated, contralateral defects (*p* = 0.02, Figures 7 and 8, and Supplemental Table  4). On histological examination, the presence of a defect was confirmed in all specimens at 7 days but the defect was filled with a hematoma and fibrin deposition only in treated defects (Figure 9). No evidence of foreign body reaction was identified. Nuclear density seemed generally increased, especially in treated defects. Overall, histological scores increased from 7 to 28 days: angiogenesis and collagen scores did not differ between time periods, but cartilage formation increased between 7 (none observed) and 28 days (0.8 ± 0.8, *p* = 0.001). However, no difference was detected between groups at any evaluation time (Figure 9 and Supplemental Table  4).

Total histological scores at 28 days correlated positively with cross section areas of tendons and negatively with the elastic modulus and stiffness of tendons (*p* = 0.026). Spearman's correlation coefficients ranged between 0.45 and 0.59. No correlation was found between fluorescence at 28 days and biomechanical properties of tendons (*p* ≥ 0.4).

## 4. Discussion

Chitosan has been found to induce the formation of spheroids by stem cells, a mechanism proposed to enhance their stemness [25, 26]. Meanwhile, several publications support hypoxic preconditioning as a strategy to enhance multilineage differentiation and survival of stem cells after* in vivo* implantation [28, 34]. A couple of publications report encouraging results when both strategies are combined to influence adult tissue-derived stem cells [35, 36]. However, the combined effects of hypoxia and chitosan conditioning have not previously been tested on UCM-MSCs. The main findings of our study are as follows: (1) conditioning eqUCM-MSCs with chitosan under hypoxia affected the DNA content but improved the expression of stemness genes after 7 days of exposure, compared to standard culture conditions; (2) eqUCM-MSCs survived and remained present in tendon defects 28 days after implantation; (3) histological scores of all tendons improved between days 7 and 28 and did not differ between groups; (4) tendons treated with conditioned cells had smaller cross areas and superior tensile properties at 28 days than tendons treated with standard eqUCM-MSCs.

The cellularity of conditioned cultures was approximately 72% lower than that of standard cultures. This finding was based on DNA content, rather than serial cell counts, to accommodate the formation of spheroids on chitosan. The lower DNA content measured at day 3 in conditioned cells is consistent with a poor attachment of cells to the substrate. We have previously reported on the limited attachment of chondrocytes and bone marrow stem cells, when seeded on chitosan sponges or fibers [18, 20, 24, 37]. This lack of cell attachment was attributed to the chemical composition, rather than the structural characteristics of the support [22, 24]. The chitosan used in our study was specifically selected for its high degree of deacetylation, a characteristic expected to encourage cell adhesion [24]. However, other factors such as surface hydrophobicity [38] and the presence of adhesion peptides along the surface of the matrix [39] can affect cell adhesion. Some evidence suggests that chitosan may improve cell proliferation [40, 41] and the DNA change between days 3 and 7 was similar between conditioned and standard cells in our study. It is therefore unlikely that the lower cellularity in conditioned cells at day 7 could be attributed to a negative effect of chitosan beyond the initial cell attachment phase.

Our results provide evidence that exposure for 7 days to chitosan and hypoxia improved the stemness of eqUCM-MSCs which is consistent with previous reports. The mechanism by which chitosan enhances the stemness of MSCs remains unclear but has been linked to formation of spheroids (cell aggregates) rather than a monolayer arrangement of cells in contact with chitosan. Huang et al. [26] found that CD44 blockage prevented the formation of spheroids and affected the expression of genes related to stemness, attributing a role to the Rho/Rho-associated kinase signaling pathway. Spheroid formation may also influence the stemness of MSCs by generating a relatively hypoxic microenvironment [25]. Although the majority of studies reporting on beneficial effects of hypoxia have focused on bone marrow-derived cells [26], a few studies have confirmed these effects on fetal adnexa-derived MSCs [29–31]. Transcriptional responses to hypoxia are mediated by hypoxia inducible factors, and increased mRNA expression levels of HIF1*α* and HIF-2*α* were reported in human UCM-MSCs [29, 42]. These factors are known to regulate the expression of several cell cycle molecules, including the Notch signaling pathway, and have been proposed to be responsible for improving multilineage differentiation of stem cells.

The tendon injury model selected here is designed to provide preliminary results prior to clinical trials in horses, as natural animal disease models of human tendinopathies. Indeed, the morbidity and cost associated with equine models justify this initial use of rodent models. The patellar tendon in rats is large enough to allow the creation of a defect without subsequent rupture. The bilateral patellar tendon defect used here limits interindividual variation via creation of an internal control in each rat. The absence of fluorescence in untreated tendon defects confirms the absence of migration of stem cells from contralateral injection sites. This finding therefore validates the use of untreated tendon defects as representative of the healing that would result from conservative treatment alone.

The improvement in DNA content and stemness observed after 7 days compared to 3 days of exposure to chitosan and hypoxia prompted us to select this group for* in vivo* testing. The presence of fluorescence in both groups of treated defects throughout the study confirms the survival and integration of implanted stem cells within the defects. The presence of a hematoma in all treated defects at 7 days after surgery is consistent with the implantation of cells in ACP. All cells in our study were delivered in a clot of ACP, which may have contributed to their local retention [43]. This agent was selected because of its common use as a carrier for stem cells, accessibility, and current clinical application in equine tendinopathies [43–45]. The percentage of fluorescence did not differ between defects treated with conditioned or standard stem cells. This result prevents us from confirming previous studies reporting improved survival induced by hypoxic conditioning of stem cells [25, 28]. ACP has been used as a growth factor for stem cells and may have mitigated the negative effects that a hypoxic environment may have on standard cells [46, 47]. Platelet-derived products have also been found to improve tendon healing [48, 49]. The improvement in tensile properties in all treated tendons compared to their untreated matched controls could therefore be attributed to the combined effects of ACP and stem cells. However, ACP cannot account for the differences between tendons treated with standard versus conditioned cells in terms of healing since all cells in our study were delivered with the same amount of ACP. Instead, the greater modulus of tendons treated with conditioned compared to standard cells reflects the effects of cell exposure to chitosan and hypoxia. In the absence of difference in the fluorescence of treated sites, this improvement in tensile properties may be more likely due to improved properties of conditioned stem cells than an actual difference in the number of cells present within the defects. Since we are the first to study the influence of MSCs conditioned with chitosan and hypoxia on tendon healing, further experiments will be warranted to investigate the mechanisms behind our observation.

Biomechanical properties of treated tendons improved independently of their histological scores. The scoring system was selected for its reproducibility and correlation (negative) with biomechanical properties of tendons [33]. The score integrates factors that do not translate into biomechanical strength, such as disorganization of collagen fibers, angiogenesis, and chondrogenesis. Our scoring system may be more representative of tissue reaction and early repair, rather than remodeling, explaining its correlation with the cross area of specimen. Variability and type II error could explain the lack of difference in histological scores between treatment groups, when in fact tendons treated with conditioned cells had smaller cross areas than untreated tendons or those treated with standard cells.

## 5. Conclusions

Conditioning eqUCM-MSCs on chitosan under hypoxia appears to affect the cell yield, which may reflect poor cell attachment to chitosan film combined with decreased proliferation due to hypoxia. However, chitosan and hypoxia improved the stemness of eqUCM-MSCs and their contribution to the healing of tendon defects. Poor cell yield is critical in autogenous cell therapies, where donor cell use must be optimized. This limitation is less relevant in allogenic cell therapies, especially since eqUCM-MSCs are abundant and easy to expand. Nonetheless, strategies may be considered in the future to improve initial cell attachment to chitosan, such as coating films with collagen [19, 20], or crosslinking chitosan with other agents [41].

## Supplementary Material

Supplemental data include tables listing means, standard deviations, and statistical differences corresponding to the graphs in Figures 3, 4, 6, and 7.
