# Supplementary material for: Effects of Hypoxia and Chitosan on Equine Umbilical Cord-Derived Mesenchymal Stem Cells
Source: Stem Cells Int. 2016 Jun 9;2016:2987140. doi: 10.1155/2016/2987140 (PMC4917753; doi:10.1155/2016/2987140)
Supplement: Supplementary file 1 — Supplemental data include tables listing means, standard deviations, and statistical differences corresponding to the graphs in Figures 3, 4, 6, and 7. [file 2987140.f1.pdf]

Supplemental data:

Table 1: *In vitro* comparison of cells cultured under standard conditions (S) or conditioned on chitosan (C) under hypoxia. Results are expressed as means and SD (in parentheses).

Parameter	Standard cells Day 3	Conditioned cells Day 3	P value Day 3	Standard cells Day 7	Conditioned cells Day 7	P value Day 7
DNA	404.1 (51.4)	102.7 (7.0)	0.002*	783.6 (93.1)	218.3 (13.3)	0.002*
Sox2	1 (0.0)	1.26 (1.77)	0.180	1.20 (0.26)	6.07 (3.36)	0.025*
Oct4	1 (0.0)	1.04 (1.16)	0.180	1.20 (0.28)	3.40 (1.46)	0.025*
Nanog	1 (0.0)	1.09 (0.81)	0.655	0.93 (0.39)	9.40 (8.34)	0.025*

Difference within pairs was calculated as the percentage change between C and S. * indicates statistical significance at the 0.05 level of significance.

Table 2: Magnitude of change (mean ± SD) in DNA content and gene expression within each group between day 3 and day 7

	Standard cells	Conditioned cells	P value
DNA Content	1.00 ± 0.46 (p=0.002)#	1.14 ± 0.23 (p=0.002)#	0.527
Sox2	0.20 ± 0.26 (p=0.18)	15.06 ± 15.05 (p=0.025)#	0.025*
Oct4	0.20 ± 0.28 (p=0.18)	5.36 ± 4.56 (p=0.025)#	0.046*
Nanog	-0.07 ± 2.53 (p=0.655)	11.37 ± 9.62 (p=0.025)#	0.025*

#denotes p-value < 0.05 between day 3 and 7 within each group.

*denotes a significant difference (p-value < 0.05) between cells exposed to chitosan and hypoxia compared to standard conditions.

Table 3: Tensile properties of tendons treated with cells cultured under standard conditions (S, N=6) or cells conditioned with chitosan and hypoxia (C, N=6) at 28 days. Results are standardized to contralateral tendons with empty defects (E, N=12) and expressed as means and SD (in parentheses). Difference between paired S and E was calculated as the percentage change between S and E. Difference between paired C and E was calculated as the percentage change between C and E.* indicates significance at the 0.05 level of significance, based on a paired t-test comparing the magnitude of change between C and E to that between S and E, within pairs of tendons treated with cells from the same horse. # denotes a statistical difference between

treated tendons and their matched untreated tendons.						
Parameter	Tendons with Empty defects	Tendons with Standard cells	Difference between paired S and E tendons, %	Tendons with Conditioned cells	Difference between paired C and E tendons, %	P value (C-S)
Length (mm)	7.01 (0.49)	7.03 (0.41)	1.67 (5.88)	6.94 (0.57)	-2.22 (4.53)	0.23
Cross area (mm^2)	14.49 (3.27)	14.95 (4.14)	23.70 (48.11)	11.99 (3.41)	-26.54 (17.27)[#]	0.04*
Modulus (Mpa)	2.28 (1.33)	2.92 (1.27)	34.53 (88.89)	4.49 (3.20)	176.60 (223.06)[#]	0.04*
Relaxation time (s)	13.96 (2.59)	13.42 (3.11)	-11.69 (19.56)	14.33 (1.75)	18.65 (33.18)	0.08
Stiffness (N/mm)	4.31 (1.84)	5.78 (1.94)	36.45 (68.43)	7.29 (5.15)	98.96 (120.87)	0.11
Hysteresis (%)	0.29 (0.19)	0.34 (0.17)	173.47 (421.68)	0.22 (0.19)	-3.45 (61.96)	0.38

Table 4: Histological features of tendons treated with cells cultured under standard conditions (S) or cells conditioned with chitosan and hypoxia (C) at 7 and 28 days, standardized to contralateral tendons with empty defects (E) and expressed as means and SD (in parentheses). Results at 28 days are based on sample size N = 12, 6, and 6 for E, S, and C respectively. Results at 7 days are based on sample size N = 8, 4, and 4 for E, S, and C respectively. Difference between paired S and E was calculated as the percentage change between S and E. Difference between paired C and E was calculated as the percentage change between C and E. The P value is based on a Wilcoxon signed-rank test comparing the magnitude of change between C and E to that between S and E, within pairs of tendons treated with cells from the same horse.

Parameter	Tendons with Empty defects	Tendons with Standard cells	Difference between paired S and E tendons, %	Tendons with Conditioned cells	Difference between paired C and E tendons, %	P value (C-S)
Fluorescence at day 7 (% area)	12.74 (15.23)	42.30 (8.60)	2,070.30 (3,073.08)	19.12 (14.48)	227.41 (253.86)	0.69
Histological score at day 7	3.05 (0.71)	3.17 (0.33)	0.89 (21.89)	3.44 (0.43)	25.71 (33.32)	0.19
Fluorescence at day 28 (% area)	1.92 (1.72)	6.53 (4.00)	427.51 (767.75)	4.74 (1.77)	389.84 (213.59)	0.72
Histological score at day 28	4.27 (1.10)	4.31 (1.04)	16.87 (48.14)	3.88 (1.08)	-14.05 (16.70)	0.11
